# Supplementary material for: A new approach for increasing graduate students’ science communication capacity and confidence
Source: PeerJ. 2024 Dec 20;12:e18594. doi: 10.7717/peerj.18594 (PMC11665425; doi:10.7717/peerj.18594)
Supplement: Supplemental Information 2 — Pre- and Post-Surveys, Daly-Miller Writing Apprehension Test, Tutoring Philosophy assignment for tutor training, and Writing Assistant Summer Training Schedule. [file peerj-12-18594-s002.pdf]

SciWrite: PRE-WORKSHOP SURVEY **Email Address** ______________________________ **Date** ____________

1. Indicate the degree you are working towards. (Please circle just one). MA MS MESM PhD

2. What year are you? (Please circle just one.) 1st 2nd 3rd 4th 5th 6th 7th 8th+

3. Indicate your primary department. _________________

As a NSF-funded program, we are asked to collect the following demographic data about our participants, although your responses are optional.

4. Gender: Male Female
5. Race: American Indian/Alaskan Native Asian Native Hawaiian or Other Pacific Islander Black or African American White
6. Ethnicity: Hispanic or Latino Not Hispanic or Latino

7. How did you hear about the workshop? ____
____

8. What is the primary reason you are attending today's workshop? ______________________________________
____

9. What is your level of confidence as a writer? not confident 1 2 3 4 5 quite confident

10. What is your level of confidence as a scientist? not confident 1 2 3 4 5 quite confident

On writing projects during the last year, how often have you:

	Never	***Seldom***	***Sometimes***	***Frequently***	***Always***
11. Engaged in any informal discussion with a peer or advisor that led to revision of your writing?					
12. Shared your unfinished writing with a peer or advisor?					
13. Been asked inside or outside a class to review a peer's writing?					
14. Engaged in any structured discussion or review that led to revision of you writing?					
15. Created multiple drafts of a single project?					
16. Produced an outline prior to starting a writing assignment?					
17. Ensured that all paragraphs have topic sentences?					

18. What is your level of confidence in being able to write an effective introduction for a scientific paper?
Not confident 1 2 3 4 5 Quite confident

19. As a graduate student, have you created a piece of writing related to your academic work that was intended for a non-specialist or public audience? Yes No

When you are writing, how would you describe your:

20. Regular routine? Inconsistent 1 2 3 4 5 Consistent
21. Preferred environment? Random 1 2 3 4 5 Established

SciWrite: NRT-IGE: POST-WORKSHOP SURVEY

1. URI E-mail address ______________________________________

2. What was the most useful thing you learned at today's workshop?

3. What was the least useful aspect of today's workshop?

4. What did you learn today that surprised you?

5. What aspects of writing would you like future workshops to cover?

6. How might your piece of writing change based on what you learned today?

7. How useful was this workshop for the writing you do as a graduate student?
not very useful 1 2 3 4 5 very useful

8. How confident do you feel about the training or practice you gained in this workshop?
not confident 1 2 3 4 5 very confident

9. How likely would you be to recommend this workshop to a friend or colleague?
not likely 1 2 3 4 5 very likely

As a result of attending the SciWrite workshop, do you expect to do any of the following?

	Definitely Not	***Probably Not***	***Not Sure***	***Maybe***	***Definitely***
10. Share a future unfinished piece of writing with a peer or colleague?					
11. Create or maintain a consistent writing routine?					
12. Create or maintain an established writing environment?					
13. Use outlining or topic sentences to organize future writing projects?					
14. Create multiple drafts of a single project?					
15. Share your scientific research with a public audience?					

16. What is your level of confidence as a writer? not confident 1 2 3 4 5 quite confident

17. What is your level of confidence as a scientist? not confident 1 2 3 4 5 quite confident

The Daly-Miller Test

E-mail address: ____

Are you a SciWrite@URI fellow? ___ Yes ___ No ___ Uncertain

Select the response from 1 to 5 that best suits your feelings about the following statements.

Remember: There are no correct answers, only give your honest response to each item. Thank you for your participation and send me your comments!

----
5 Strongly Disagree - 4 Disagree - 3 Uncertain - 2 Agree - 1 Strongly Agree
----

1. I avoid writing. (+) 5 4 3 2 1

2. I have no fear of my writing's being evaluated. (-) 5 4 3 2 1

3. I look forward to writing down my ideas. (-) 5 4 3 2 1

4. I am afraid of writing essays when I know they will be evaluated. (+) 5 4 3 2 1

5. Taking a composition course is a very frightening experience.(+) 5 4 3 2 1

6. Handing in a composition makes me feel good. (-) 5 4 3 2 1

7. My mind seems to go blank when I start to work on my composition. (+) 5 4 3 2 1

8. Expressing ideas through writing seems to be a waste of time. (+) 5 4 3 2 1

9. I would enjoy submitting my writing to magazines for evaluation and publication.(-) 5 4 3 2 1

10. I like to write down my ideas. (-) 5 4 3 2 1

11. I feel confident in my ability to express my ideas clearly in writing. (-) 5 4 3 2 1

12. I like to have my friends read what I have written. (-) 5 4 3 2 1

13. I'm nervous about writing. (+) 5 4 3 2 1

14. People seem to enjoy what I write. (-) 5 4 3 2 1

15. I enjoy writing. (-) 5 4 3 2 1

16. I never seem to be able to write down my ideas clearly. (+) 5 4 3 2 1

17. Writing is a lot of fun.(-) 5 4 3 2 1

18. I expect to do poorly in composition classes even before I enter them. (+)   5   4   3   2   1

19. I like seeing my thoughts on paper. (-)   5   4   3   2   1

20. Discussing my writing with others is enjoyable. (-)   5   4   3   2   1

21. I have a terrible time organizing my ideas in a composition course. (+)   5   4   3   2   1

22. When I hand in a composition, I know I'm going to do poorly. (+)   5   4   3   2   1

23. It's easy for me to write good compositions. (-)   5   4   3   2   1

24. I don't think I write as well as most other people. (+)   5   4   3   2   1

25. I don't like my compositions to be evaluated. (+)   5   4   3   2   1

26. I'm not good at writing. (+)   5   4   3   2   1

Tutoring Philosophy

Assignment: Write a 2-3 page essay that outlines your personal tutoring philosophy. You should use a combination of analysis and synthesis of texts we have read, your thoughts and contributions from previous discussions, and your own personal experiences.

Directions: Now that you've read about and discussed many theoretical and philosophical frameworks of Writing Center pedagogy, please answer the question: **"What is your personal tutoring philosophy?"** Refer to various readings and discussions from training, and don't forget to incorporate terminology and concepts that we've learned so far this term to demonstrate your learning process.

Purpose: A tutoring philosophy is a statement of your beliefs, assumptions, and knowledge about tutoring; this statement will likely depend on your personal experiences, what you have learned about pedagogy so far in school, and what you have learned during this training. In simplistic terms, it all boils down to: **"This is what I believe as a writing tutor."**

There are multiple benefits of writing a tutoring philosophy. This will be your first opportunity to think deeply about the tutoring framework(s) that you will utilize in each session. Such reflection will help you feel more prepared for your WC sessions. If you continue on as a Writing Assistant in the future, reflecting on and building upon this tutoring philosophy will help you understand *why* you do what you do during a WC session. Finally, if you decide to pursue tutoring and/or teaching as a future career, having an already developed philosophy will help add credibility to your CV and will give you an advantage during job interviews.

Process:

GETTING STARTED: You will want to read through the tutoring philosophy examples we have provided on Sakai, and use these examples as a model for how to approach your own essay.

WHAT TO CONSIDER: Your tutoring philosophy might discuss the practices and methods you hope to employ in sessions to help writers learn (e.g., both facilitative and directive strategies, specific types of scaffolding, rhetorical analysis, etc.). Additionally, it would be beneficial to give specific examples of what you have learned so far from training, and/or what you have learned from your own life experiences. It could include a series of position statements that address specific philosophies we have mentioned during

training so far, including the importance of the facilitative process, students' rights to their own voice and ideas, the necessity of facilitating productive diversity in a session, focusing on process rather than product, and many others that we may not have time to address this summer.

*Some concepts from this essay assignment borrowed from Susan Spangler's English 455 class, SUNY university

Writing Assistant Summer Training Schedule

Weekly Topics	Assignments
May 3: Introduction to facilitative questions	• *LG*: Ch. 3 The Tutoring Process<br>• Retreat workshop: practicing facilitative questions
Week 1<br>May 22-26: Writing Center theory and pedagogy	• *LG*: Ch.1 Why We Tutor<br>• **DUE:** Online self-reflection
Week 2<br>May 29-June 2: Writing Center theory and pedagogy, Peer-to-peer framework	• *LG*: CH. 3 (if you haven't yet read it)<br>• Turabian, "What research is"<br>• **DUE:** Scenario One online discussion: brainstorming helpful tutoring strategies
Week 3<br>June 5-9: Finding a balance between facilitative and directive strategies	• *LG*: Ch. 6 Tutoring Practice<br>• Dinitz & Harrington, "The Role of Disciplinary Expertise in Shaping Writing Tutorials"
Week 4<br>June 12-16: Practice Session	• Undergraduate sample paper<br>• **DUE:** Online discussion of sample paper with three strengths and three areas for improvement
Week 5<br>June 19-23: Practice Session	• *LG*: Ch. 4 Examining expectations<br>• **DUE:** First sample paper and discussion
Week 6<br>June 26-30: Writing Process as a framework for tutoring	• *LG*: Ch. 2 The Writing Process
Week 7<br>July 3-7: Practice Session	• Supplemental: Tauber (excerpt: pgs. 641–653: 'Writing Consulting at the Graduate Level' and 'A Portrait of the Compositionist as an Independent Consultant')<br>• **DUE:** Second sample paper and discussion
Week 8<br>July 10-14: Multiple genres, genre theory	• Babcock & Thonus, "Chapter 5: Tutoring Activities" (excerpt: pgs. 111–117: 'Speaking; Listening; Reading')<br>• Supplemental: Garbus, "Tutoring Graduate Students in the Writing Center"

	• **DUE:** Babcock & Thonus discussion
Week 9<br>July 17-21: Multiple genres, genre theory	• Carter, "Ways of Knowing, Doing, and Writing in the Disciplines" (excerpt: pgs. 388-393) Skim the rest, paying close attention to the lettered lists of outcomes<br>• **DUE:** Third sample paper and discussion
Week 10<br>July 24-28: Multiple genres, genre theory	• Hall and Sarmiento: IWCA Genre Presentations and Genre Card Activity (Genre Cards, Audience and Purpose Sheet)<br>• Supplemental: Kohn, "Can They Tutor Science? Using Faculty Input, Genre, and WAC-WID to Introduce Tutors to Scientific Realities"<br>• **DUE:** Genre Card Activity
Week 11<br>Aug. 1-4: Tutoring Philosophy	• *LG*: Ch. 8 Reading in the Writing Center<br>• Work on Tutoring Philosophy draft<br>• **DUE**: Scenario Two
Week 12<br>Aug. 7-11: Observing WC sessions	• Ch. 5 Observing in the Writing Center<br>• **DUE:** Eli review of Tutoring Philosophy draft
Week 13<br>Aug. 14-18: Tutoring Philosophy	• **DUE:** Final Draft of Tutoring Philosophy
Week 14<br>Aug. 21-25: Practice Session	• Ch. 7 Reflecting on the first session<br>• **DUE:** Mock Writing Center session reflection
**September:** In-person practice sessions!	
Sep. 4-15	• Observations at undergraduate Writing Center
Sep. 18-22	• Team sessions
Sep. 25-29	• Session with Erin
October	• Debriefing with Erin. Self-reflection
