# Supplementary material for: A new approach for increasing graduate students’ science communication capacity and confidence
Source: PeerJ. 2024 Dec 20;12:e18594. doi: 10.7717/peerj.18594 (PMC11665425; doi:10.7717/peerj.18594)
Supplement: Supplemental Information 3 — Outreach document composed by a SciWrite Fellow for their Science Communication internship. [file peerj-12-18594-s003.pdf]

LOCATION

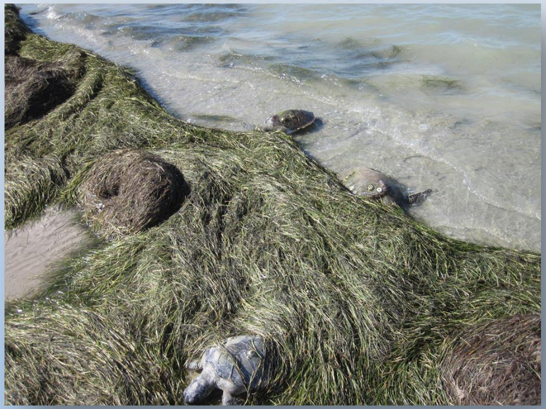

Green turtles stranded in sea grass.

Although sea turtles tend to be seasonal residents in some parts of the world, several species can be seen in the Gulf of Mexico year-round. Summer at Padre Island is well known to sea turtle enthusiasts, but it is also important to keep an eye out for sea turtles in the winter. Juvenile green sea turtles are the most common sea turtle found along the Texas coast during the winter but sudden, dramatic drops in temperatures can put their lives at risk!

WHY ARE SEA TURTLES HERE IN THE WINTER?

Some green turtles engage in a trade-off by staying here through the winter months. While most sea turtles can seek shelter from the rapidly declining temperatures by heading to deeper, ocean waters, green turtles often risk exposure because their primary food source (sea grasses and algae) is abundant in shallow areas like Laguna Madre.

TIMING IS CRITICAL

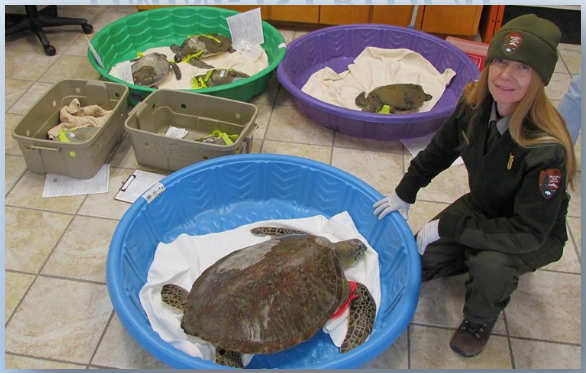

Dr. Donna Shaver with rescued turtles.

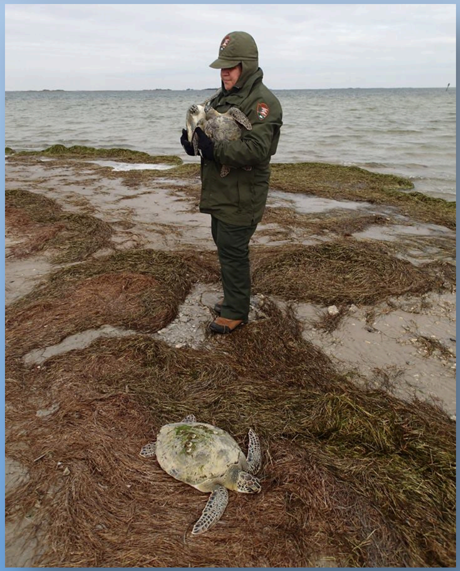

Cynthia Rubio rescuing stranded turtles.

EVERY TURTLE MATTERS

If you find a cold-stunned sea turtle IMMEDIATELY call

(361) 949-8173 Ext. 226

Padre Island National Seashore
Division of Sea Turtle
Science and Recovery
P.O. Box 181300
Corpus Christi, TX 78480

www.nps.gov/pais

Winter Watch:

Sea Turtles

Cold Stunning

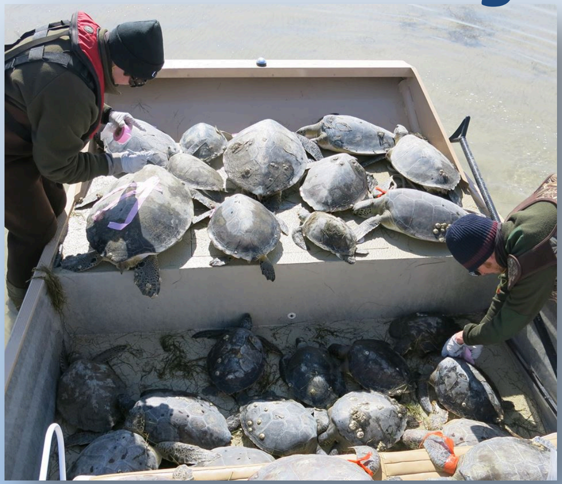

Rescuing cold-stunned turtles.

WHAT IS IT AND WHY DOES IT AFFECT SEA TURTLES?

Unlike us, sea turtles can't regulate their body temperatures and rely on the environment around them to stay warm, so when **water temperatures quickly drop**, especially after cold fronts, sea turtles often become cold stunned. Cold stunning (**severe hypothermia**) happens when their body temperatures drop dangerously low, causing them to become incapacitated. This condition arises when **water temperatures drop below 50°F**. Once cold-stunned, sea turtles are unable to swim and tend to float at the water surface. Unable to move, they are pushed through the water by the wind and can become stranded on beaches, sand bars, and islands within Laguna Madre.

Cold stunning is the most significant cause of green sea turtle strandings in Texas.

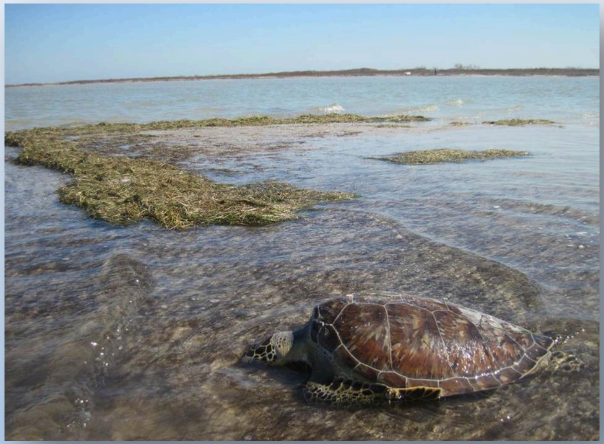

Stranded green turtle.

HOTSPOTS

The shallow waters in Laguna Madre can act as a trap to foraging green sea turtles. With only 3 direct passageways between the 130-mile stretch of Laguna Madre and the Gulf of Mexico (Packery Channel, Mansfield Channel, and Brazos Santiago Pass) and an average water depth of less than 4 feet, the Laguna Madre is a cold stunning hotspot.

The **map below** shows the distribution of sea grasses in Laguna Madre and the red box highlights one of the areas where large numbers of turtles are often found during these sudden, dramatic drops in temperature. Prevailing winds can push cold-stunned turtles to the shorelines along Laguna Madre at Padre Island National Seashore and northward, where they can be found and rescued.

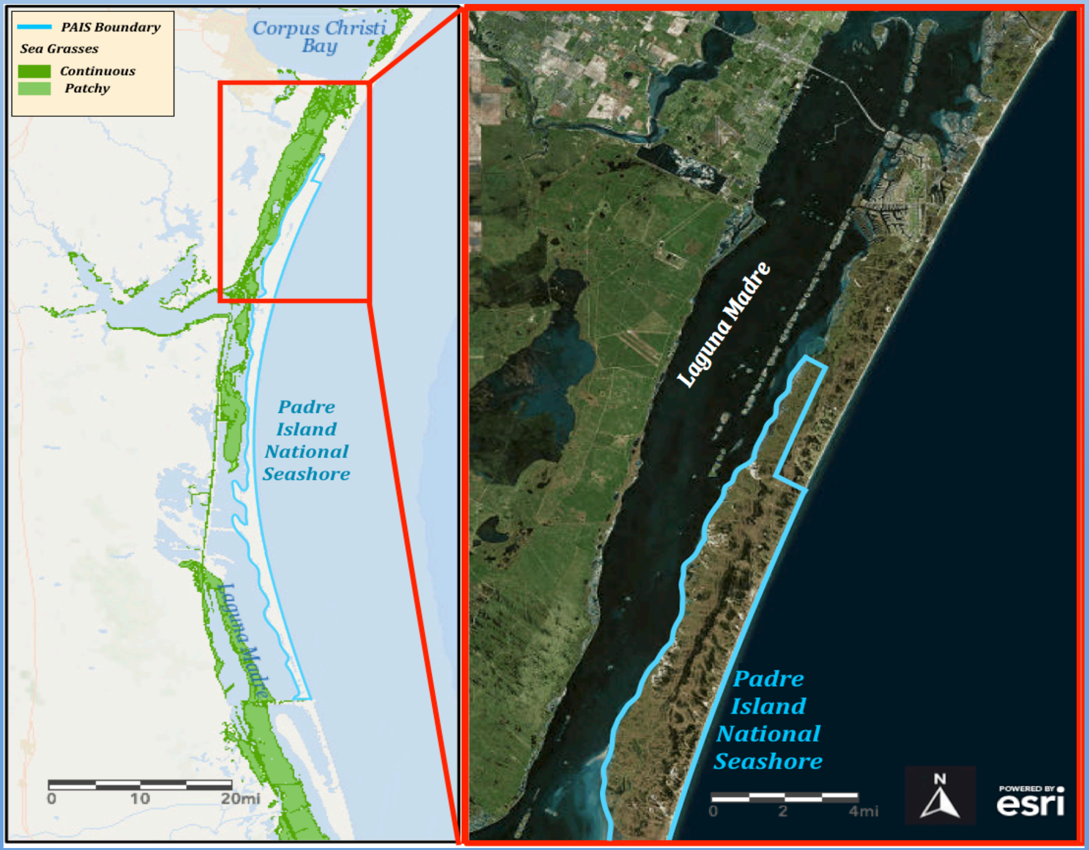

For more information, please visit: www.nps.gov/pais/learn/nature/cold-stunned-sea-turtles.htm
