# Supplementary material for: A new approach for increasing graduate students’ science communication capacity and confidence
Source: PeerJ. 2024 Dec 20;12:e18594. doi: 10.7717/peerj.18594 (PMC11665425; doi:10.7717/peerj.18594)
Supplement: Supplemental Information 4 — Outreach document composed by a SciWrite Fellow for their Science Communication internship. [file peerj-12-18594-s004.pdf]

All posts by Jessica Pita Aquino

About Jessica Pita Aquino

A Puerto Rican graduate student pursuing a Ph.D. in Biological and Environmental Sciences. Interested in research focused on Population Genetics, Ecogenomics, Global Change Biology and Infectious Diseases.

A Case of Cryptic Back-Introduction

August 21, 2018 New Research admixture, Anolis sagrei, Cayman Islands, invasive species Jessica Pita Aquino

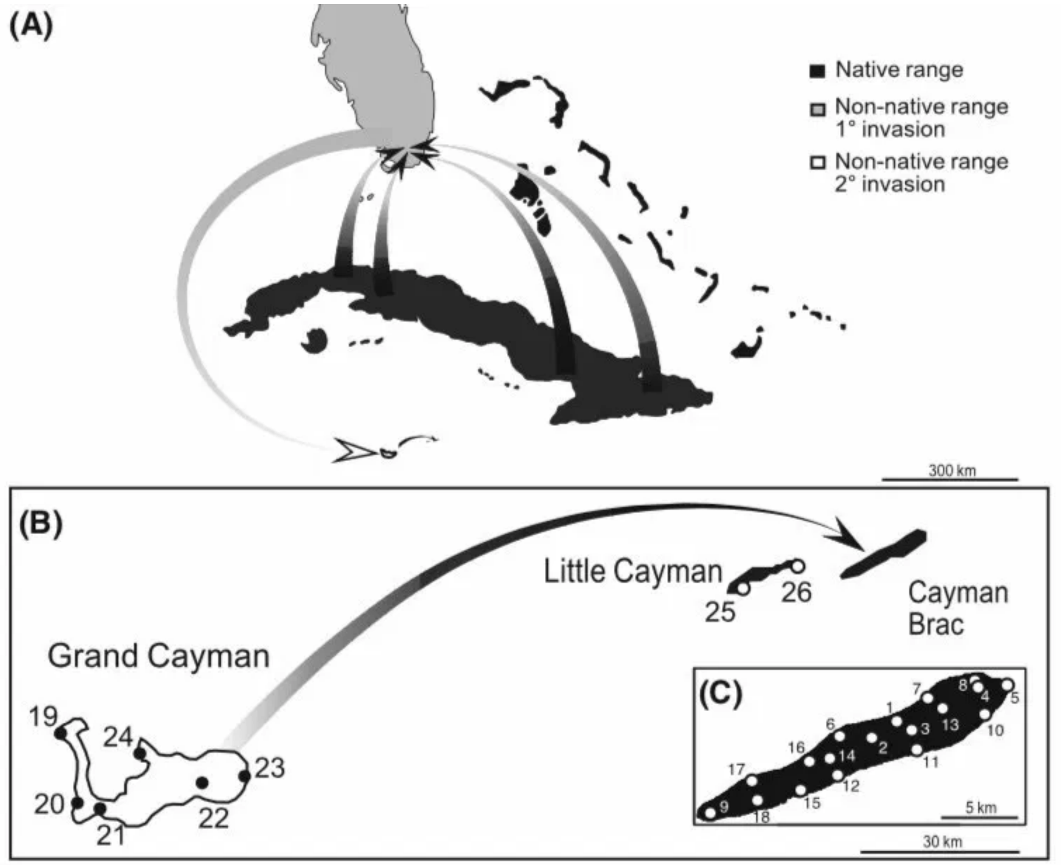

Figure 1. Native and non-native ranges of Anolis sagrei. Map from Kolbe et al. (2017).In this study, Kolbe and collaborators (2017) surveyed A. sagrei populations across Cayman Brac. First, they looked for red-dewlapped lizards to determine whether invasive A. sagrei from Grand Cayman have invaded Cayman Brac. Second, they collected brown anole lizards on Grand Cayman and Little

Cayman to determine the source of red-dewlapped A. sagrei. For all lizards captured, they quantified dewlap phenotypes (i.e., reflectance spectra) using spectrophotometric methods, measured structural habitat use (i.e., perch height and diameter) and body size (i.e., snout-vent length (SVL) and mass), and genotyped ten nuclear microsatellite loci. For lizards with intermediate multilocus genotypes or with a genotype that did not match their island, they sequenced mitochondrial DNA (mtDNA) haplotypes (ND2) to test for nuclear-mitochondrial mismatches. Genomic data was combined with previously published microsatellite genotypes (Kolbe et al. 2008) and mtDNA (ND2) sequences for the Cayman Islands (Kolbe et al. 2004, 2007). With these data, they evaluated whether invasive A. sagrei from Grand Cayman have been introduced to native populations on Cayman Brac, and if so, whether invasive lizards have interbred with native lizards.

Under current trends of globalization, human activities impact the distribution of species by facilitating dispersal of propagules. Human-mediated dispersal prevents geographic distance from being a barrier to the introduction and movement of many species. These long-distance colonization events can gather evolutionary distinct lineages that might have been separated for millions of years (e.g., Kolbe et al. 2004). Moreover, dispersal events can potentially reintroduce individuals from an invasive population back into their native range; either back into their original source population or to any part of their native range. This previously undocumented dimension of biological invasion was termed *cryptic back-introduction* by Guo (2005).

Anolis sagrei is an excellent colonist, judging by its geographical distribution. This species has reached many islands and mainland areas in the Caribbean by overwater dispersal (Williams 1969). About 2.5 million years ago, *A. sagrei* naturally colonized Cayman Brac and Little Cayman. These populations subsequently differentiated into the yellow-dewlapped endemic subspecies *A. sagrei luteosignifer* on Cayman Brac and the red-dewlapped *A. s. sagrei* on Little Cayman (Schwartz and Henderson 1991); the dewlap (i.e., an extendible flap of skin attached to the throat) is used for mate attraction, male-male and interspecific competition, and predator deterrence (Losos 2009). However, this species failed to naturally colonize the third of the Cayman Islands, Grand Cayman. In the early 1980s, through human-mediated dispersal, a red-dewlapped form of *A. sagrei* established on Grand Cayman. These populations resulted from the introduction of genetically admixed lizards from non-native populations in south Florida (Minton and Minton 1984; Kolbe et al. 2004, 2008; **Figure 1**). Since then, inter-island supply shipments by air and sea within the Caymans could have transported invasive and native brown anole lizards among the three islands. Kolbe et al. (2017) explored whether *cryptic back-introduction* is occurring in brown anole (*A. sagrei*) lizards and the implications of this type of invasion for native populations.

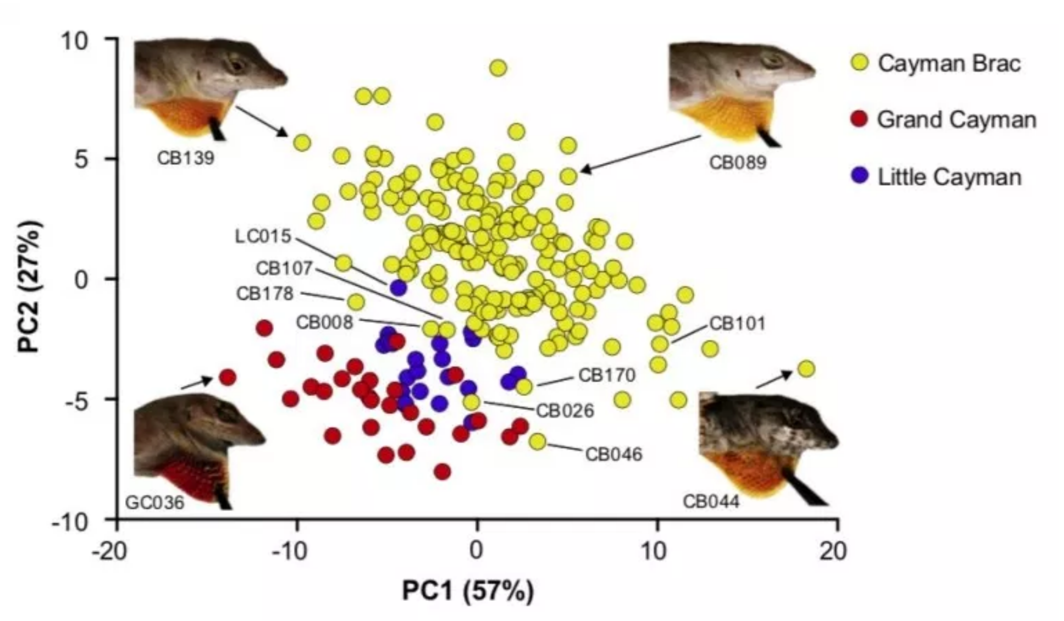

Figure 2. *Results of PCA for dewlap reflectance (Kolbe et al. 2017).*

Kolbe et al. (2017) found no differences among islands in structural habitat use. They conducted a principal component analysis (PCA) for dewlap reflectance data using the average wavelength of each lizard. PCA results show that there is strong differentiation in dewlap reflectance between yellow-dewlapped lizards on Cayman Brac and the red-dewlapped lizards on Little Cayman and Grand Cayman (**Figure 2**), which supports their field observations of red-dewlapped lizards occurring on Cayman Brac (**Figure 3B**). This suggests the introduction of brown anole lizards to Cayman Brac from either of the two other Cayman Islands.

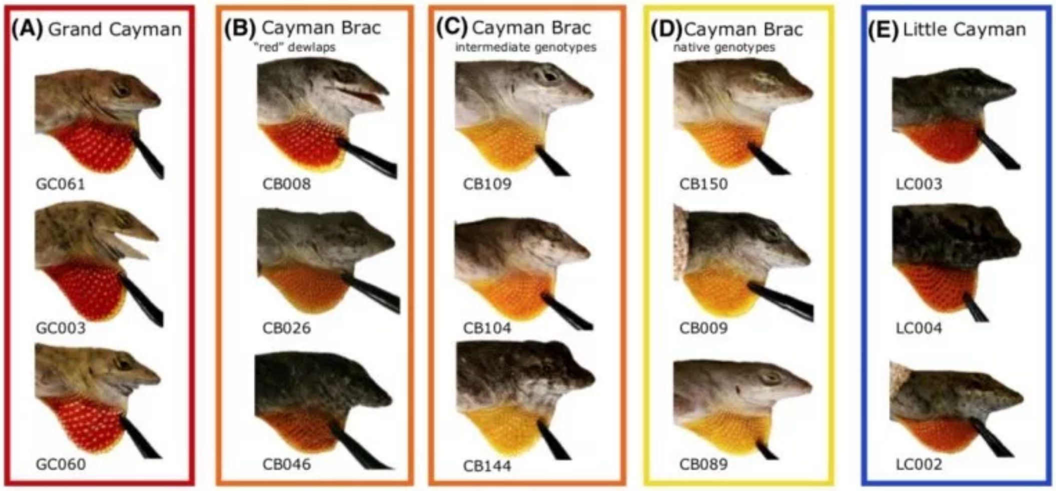

Figure 3. *Examples of Anolis sagrei dewlaps from the Cayman Islands (Kolbe et al. 2017).*

Furthermore, this study reports strong population-genetic structure among the three Cayman Islands and evidence for non-equilibrium. They identified intermediate multilocus genotypes between Grand Cayman and Cayman Brac (**Figure 4**). Also, the authors found an intermediate microsatellite genotype in one individual from Cayman Brac. This lizard had a red dewlap and a mtDNA haplotype from Grand Cayman. This mismatch among genetic and phenotypic data suggests that *A. sagrei* lizards (with different colored dewlaps) from Grand Cayman and Cayman Brac are interbreeding.

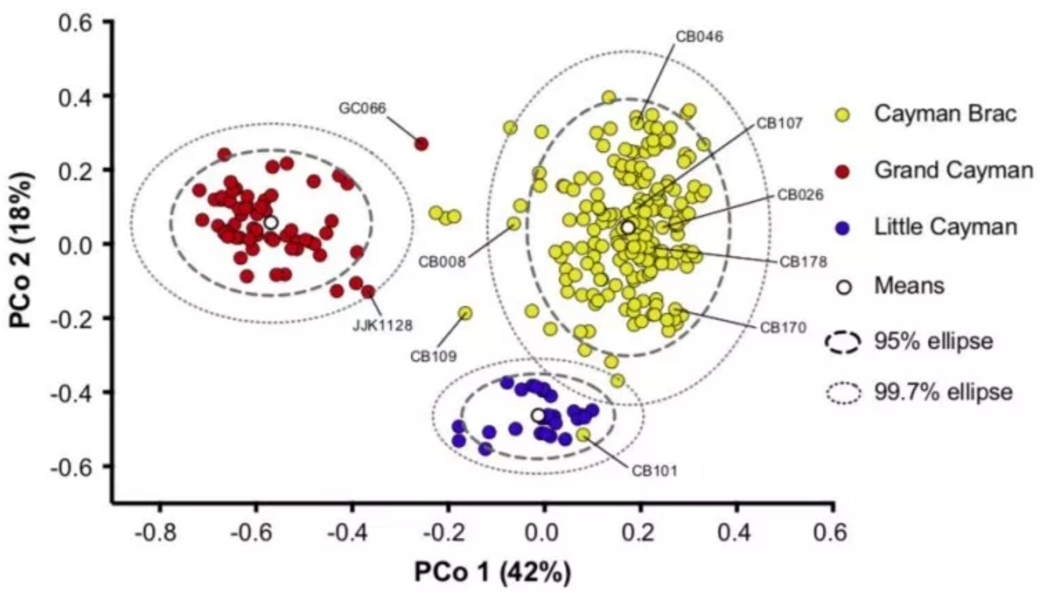

Figure 4. *Results of a PCoA using multilocus genotypes from ten microsatellite loci (Kolbe et al. 2017).*

This study reports the first evidence of cryptic back-introduction; however the frequency with which this phenomenon occurs is still unknown. By studying cryptic back-introductions we can eventually understand how lineages change though a brief period of isolation from its native range and determine if these are incompatible when brought together again. Likewise, future studies should address how phenotypic variation affects ecological interactions with native species and its consequences.

Article:

Kolbe, J. J., J. E. Wegener, Y. E. Stuart, U. Milstead, K. E. Boronow, A. S. Harrison, and J. B. Losos. 2017. An Incipient Invasion of Brown Anole Lizards (Anolis sagrei) Into Their Own Native Range in the Cayman Islands: A Case of Cryptic Back-introduction. *Biological Invasions* 19:1989–1998.

Cited Literature:

Guo, Q. 2005. Possible cryptic invasion through “back introduction”?

Kolbe, J. J., R. E. Glor, L. R. Schettino, A. C. Lara, A. Larson, and J. B. Losos. 2004. Genetic variation increases during biological invasion by a Cuban lizard. Nature 431:177–181.

Kolbe, J. J., A. Larson, and J. B. Losos. 2007. Differential admixture shapes morphological variation among invasive populations of the lizard Anolis sagrei. Molecular Ecology 16:1579–1591.

Kolbe, J. J., A. Larson, J. B. Losos, and K. de Queiroz. 2008. Admixture determines genetic diversity and population differentiation in the biological invasion of a lizard species. Biology letters 4:434–437.

Losos, J. B. 2009. Lizards in an Evolutionary Tree: Ecology and Adaptive Radiation of anoles. University of California Press.

Minton SA, Minton MR (1984) Anolis sagrei (brown anole). Herpetol Rev 15:77

Schwartz A, Henderson RW (1991) Amphibians and reptiles of the West Indies: descriptions, distributions, and natural history. University of Florida Press, Gainesville

Williams, E. E. 1969. The ecology of colonization as seen in the zoogeography of anoline lizards on small islands.

JMIH 2018: The Curious Case of Bark Anoles

July 20, 2018 New Research bark anole, Dewlap, ecological specialization, geographic isolation, hybridization, speciation Jessica Pita Aquino

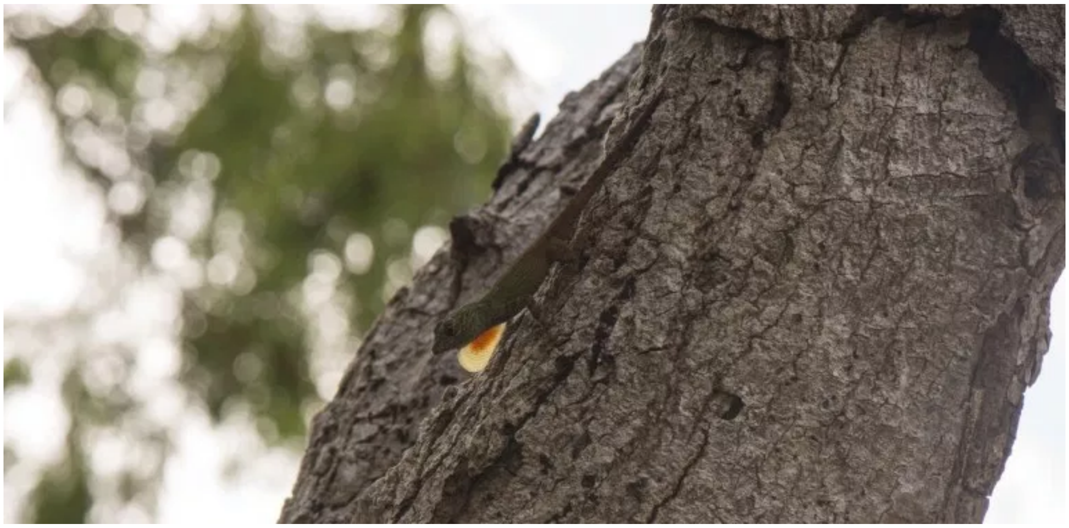

The Bark Anole (Anolis distichus ignigularis) from the Río Recodo. Photo from Richard Glor's Flickr.

The Bark Anole (*Anolis distichus*) is a highly polymorphic lizard widely distributed in Hispaniola. *Anolis distichus* is divided into 16 subspecies with dewlap colors ranging from deep wine red to pale yellow (Glor and Laport 2012). In the early days scientists, such as Albert Schwartz, argued that *A. distichus* is divisible into multiple subspecies according to an analysis of variation in body color and dewlap pigmentation. But, are they really subspecies?

During the 2018 Joint Meeting of Ichthyologists and Herpetologists (JMIH), Richard Glor shared his lab's advances on the curious case of Bark Anoles. *Anolis distichus* populations have ecological, phenotypic and genetic differences. Previous studies show a correlation between dewlap phenotype and environmental variation; in drier habitats, lizards have smaller, brighter, yellow dewlaps, while those in wetter habitats have larger, less bright, orange dewlaps (Ng et al. 2012).

Previously, the Glor Lab found strong support for the hypothesis that *A. distichus* is comprised of numerous genomically distinct populations (MacGuigan et al. 2016). Genetic divergence was associated with a biogeographic barrier, but not with dewlap color. Also, they found evidence for hybridization in contact zones with limited gene flow and intrinsic reproductive isolation between subspecies (MacGuigan et al. 2016; Ng et al. 2016). Overall, these studies suggest that geographic isolation, as well as ecological specialization, contribute to speciation.

The Glor Lab continues putting together the pieces of the puzzle. Most recently, they sequenced and assembled whole genome sequence data for *A. distichus* to identify the genomic basis for species differences and speciation.

JMIH 2018: How Does Artificial Light at Night Affect Anoles?

July 17, 2018 Introduced Anoles, New Research ALAN, artificial light, urbanization
Jessica Pita Aquino

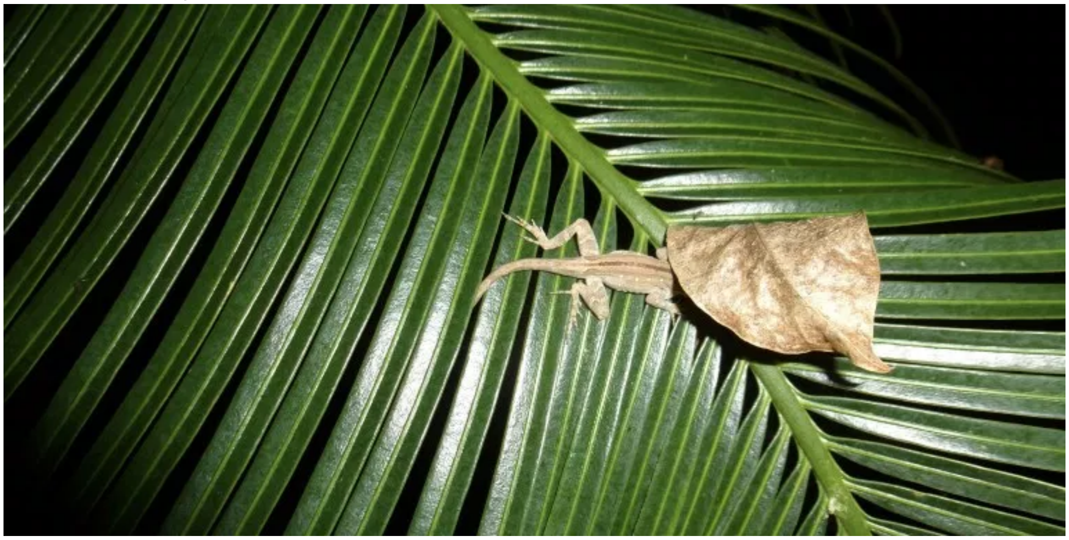

Crested Anole (Anolis cristatellus) under a leaf. Photo by Chris Thawley.

Conservation biologists have long been concerned about the effects of human development on species and environments. Urban habitats can significantly change lighting patterns for animals by increasing nocturnal ambient illumination. Artificial light at night (ALAN) has the potential to disrupt an organism's physiology, behavior, and ecology. However, light pollution remains poorly studied and is a concern for urban herpetofauna.

Anolis lizards in Miami, Florida are a great system to study the effects of ALAN on behavior, health, reproduction, and survival. Anoles are diurnal and are adapted to a distinct photic habitat appropriate to their sun/shade preferences. However, many anole species have been observed active at night where artificial lights are prevalent. So, what are the effects of ALAN on anole fitness?

Chris Thawley, a postdoctoral researcher in the Kolbe Lab at the University of Rhode Island, is interested in whether ALAN imposes selection on anoles and how they might adapt to these pressures. Chris conducted a field experiment introducing landscape lightning into a previously unlighted habitat within an urban matrix. For over two months, he assessed whether Brown Anoles (*Anolis sagrei*) and Crested Anoles (*A. cristatellus*) experienced higher levels of ALAN at their sleeping perches and if these lizards behaviorally avoided exposure to artificial light. Also, lizards were marked and followed to determine if light exposure impacted survival, growth, body condition, and physiology.

Chris found that *A. sagrei* and *A. cristatellus* lizards are not behaviorally avoiding ALAN at night. Anoles that were more exposed to artificial light had lower glucose levels compared to those that were less exposed. Also, there were no dramatic changes in reproduction, but ALAN reduced follicle size. Egg

mass showed a positive relationship with snout-vent length (SVL) in lizards exposed to ALAN, which suggests that ALAN increases egg mass in larger lizards. Chris continues analyzing growth and survival data and aims to explore if there is a correlation between levels of corticosterone (CORT), melatonin, and glucose.

JMIH 2018: Do Ecomorphs and Parasites Coevolve?

July 15, 2018 New Research bahamas, JMIH2018, parasitology Jessica Pita Aquino

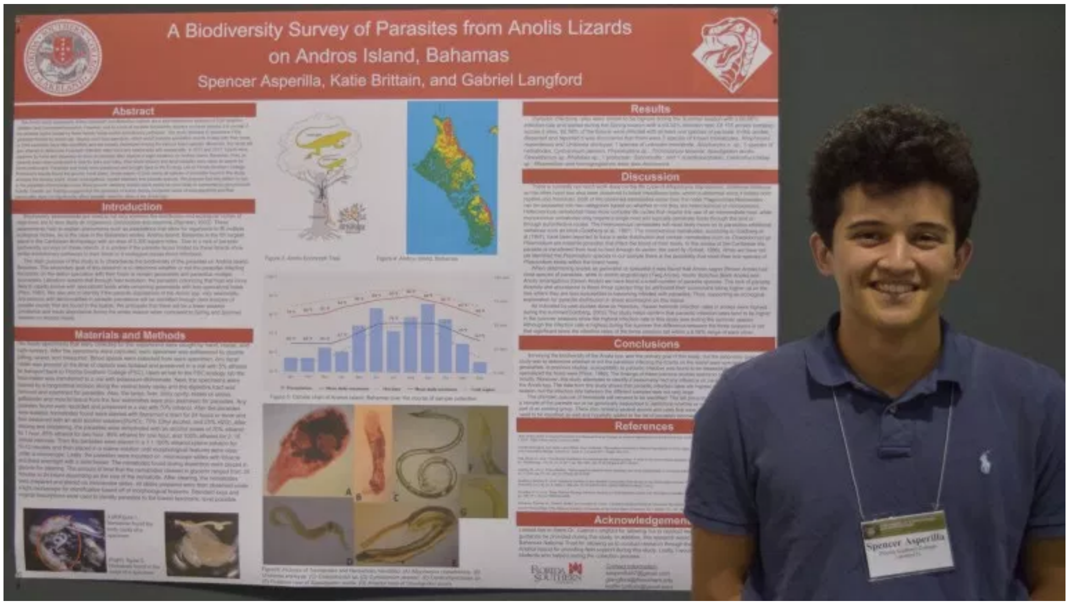

Spencer Asperilla presenting his poster, "A Biodiversity Survey of Parasites from Anolis Lizards on Andros Island, Bahamas", at the 2018 Joint Meeting of Ichthyologists and Herpetologists (JMIH).

Anolis species inhabiting the Caribbean provide a great example of adaptive radiation and convergent evolution in ecology, morphology, and behavior. Adaptation, diversification, and specialization to a particular microhabitat and dietary resource, created a great diversity among anoles. But what about their parasite assemblages? Andros Island in the Bahamas is the fifth largest island in the Caribbean Archipelago. However, it is still unclear if the parasite fauna hosted by *Anolis* lizards show similar evolutionary pathways.

In 2016, after an amazing experience studying abroad at ForFar Field Station on Andros Island, Spencer Asperilla and Katie Brittain joined the Langford Lab at Florida Southern College. Spencer and Katie were interested in documenting parasite species present in Bahamian *Anolis* lizards to determine if these are specialists or generalists among ecomorphs and identify if parasite populations vary seasonally. They conducted parasite biodiversity surveys on three sites on Andros Island, which involved capturing lizards and collecting blood smears and fecal matter. Specimens and samples were transported to Florida Southern College where they were processed and analyzed for parasites.

Spencer and collaborators found that parasitic infection rate was highest during the Summer (66.66%), and lowest during the Spring (60.56%); however these differences were not significant. Climate variables, such as mean daily temperature and precipitation, were evaluated, but no seasonal pattern could be determined for parasite infections in Bahamian lizards. As for parasite diversity, Brown Anole (*Anolis sagrei*; trunk-ground ecomorph) lizards had most species of parasites present, while *A. angusticeps* (twig ecomorph), *A. distichus* (Bark Anole; trunk ecomorph), and *A. smaragdinus* (Green Anole; trunk-crown ecomorph) had lower species diversity. The authors suggest these differences are related to the biology of the different ecomorphs. Trunk-ground anoles, such as *A. sagrei*, might be more susceptible to parasite infection by descending to the ground to capture prey or interact with a conspecific, whereas the other ecomorphs remain higher up in the tree. Ground-dwelling insects may serve as intermediate hosts for parasites found in trunk-ground anoles. Spencer and collaborators propose that habitat use, as well as dietary composition, serve as an ecological explanation for parasite distribution among ecomorphs.

The big question remains unanswered: have parasite species coevolved with specific lizard hosts? The Langford Lab continues identifying parasites species to assess the diversity, host-specificity and infection patterns of Bahamian *Anolis* lizards. Spencer wants to resume this project as part of his master's thesis and he looks forward to traveling back to Andros Island to collect additional samples.

What's New in Anole Literature?

June 26, 2018 New Research Jessica Pita Aquino

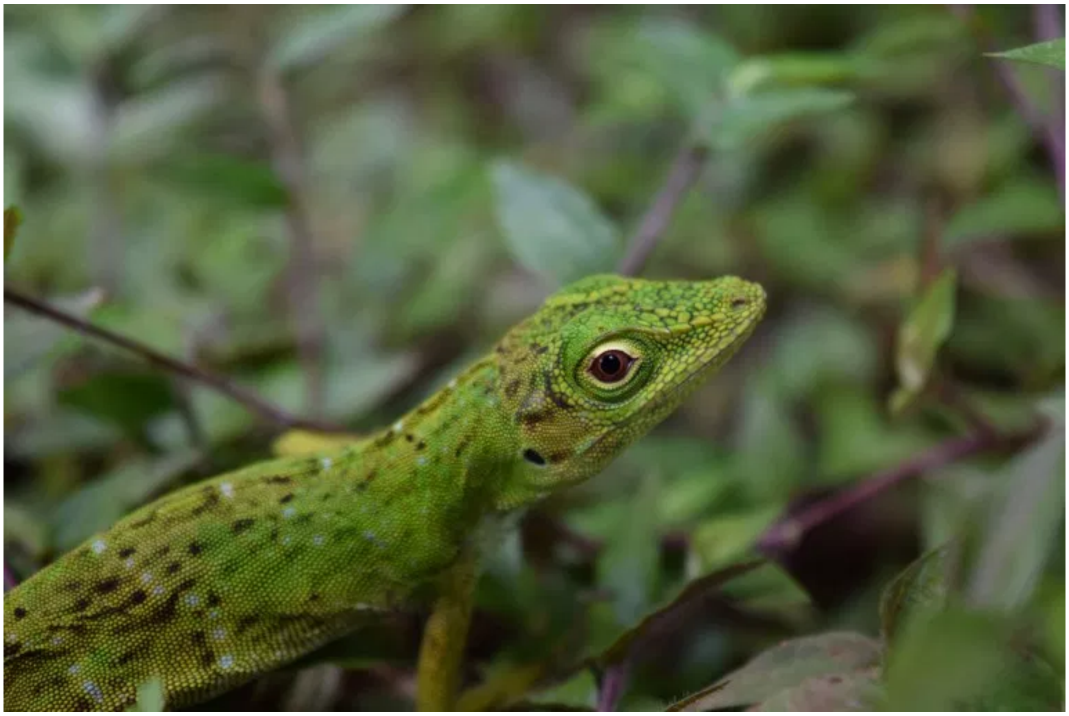

Anolis biporcatus. Photo by Jessica Pita Aquino.

Anole biologists and enthusiasts, stay updated on the latest anole research and find out about these fascinating creatures as scientists continue to make amazing discoveries! Here's what's been published in the last year and a half (2017-2018): nearly 150 papers in just a year and a half!

Anole Annals welcomes posts on new anole literature. Our goal is to try to keep the anole community up-to-date with regard to new publications, but we need help! So, if you'd like to try your hand at some science communication (or need something to list as a Broader Impact), please consider writing a post summarizing or discussing a recent paper.

And authors: there's nothing more interesting than to get the insider's view of a recent paper. Tell us the backstory: how a paper came to be and why you conducted the study in the first place. Provide the fascinating details that you can't find in a published paper. It's a great way to disseminate your work, and looks good on grant proposals.

We invite anyone interested to write posts! If you'd like to be a contributor, please write anoleannals@gmail.com.

2017

Alibardi, L. 2017. Review: Biological and Molecular Differences between Tail Regeneration and Limb Scarring in Lizard: An Inspiring Model Addressing Limb Regeneration in Amniotes. Journal of Experimental Zoology Part B: Molecular and Developmental Evolution 328:493–514.

Armstead, J. V., F. Ayala-Varela, O. Torres-Carvajal, M. J. Ryan, and S. Poe. 2017. Systematics and Ecology of *Anolis biporcatus* (Squamata: Iguanidae). Salamandra 53:285–293.

Birke, L. L., A. M. Cespedes, E. R. Schachner, and S. P. Lailvaux. 2017. Cystic Calculus in a Laboratory-housed Green Anole (*Anolis carolinensis*). Comparative Medicine 67:112–115.

Bochaton, C., S. Bailon, A. Herrel, S. Grouard, I. Ineich, A. Tresset, and R. Cornette. 2017. Human Impacts Reduce Morphological Diversity in an Insular Species of Lizard. Proceedings of the Royal Society B: Biological Sciences 284.

Bonneaud, C., I. Sepil, L. Wilfert, and R. Calsbeek. 2017. Plasmodium Infections in Natural Populations of *Anolis sagrei* Reflect Tolerance Rather Than Susceptibility. Integrative and Comparative Biology 57:352–361.

Boyer, J. F. F., and L. Swierk. 2017. Rapid Body Color Brightening is Associated with Exposure to a Stressor in an *Anolis* Lizard. Canadian Journal of Zoology 95:213–219.

02181503
