# Supplementary material for: A new approach for increasing graduate students’ science communication capacity and confidence
Source: PeerJ. 2024 Dec 20;12:e18594. doi: 10.7717/peerj.18594 (PMC11665425; doi:10.7717/peerj.18594)
Supplement: Supplemental Information 5 — Outreach document composed by a SciWrite Fellow for their Science Communication internship. [file peerj-12-18594-s005.pdf]

Celebrating 40 Years of Kemp's Ridley Sea Turtle Conservation

posted Jul 16, 2018 7:17 PM by Felicia Page

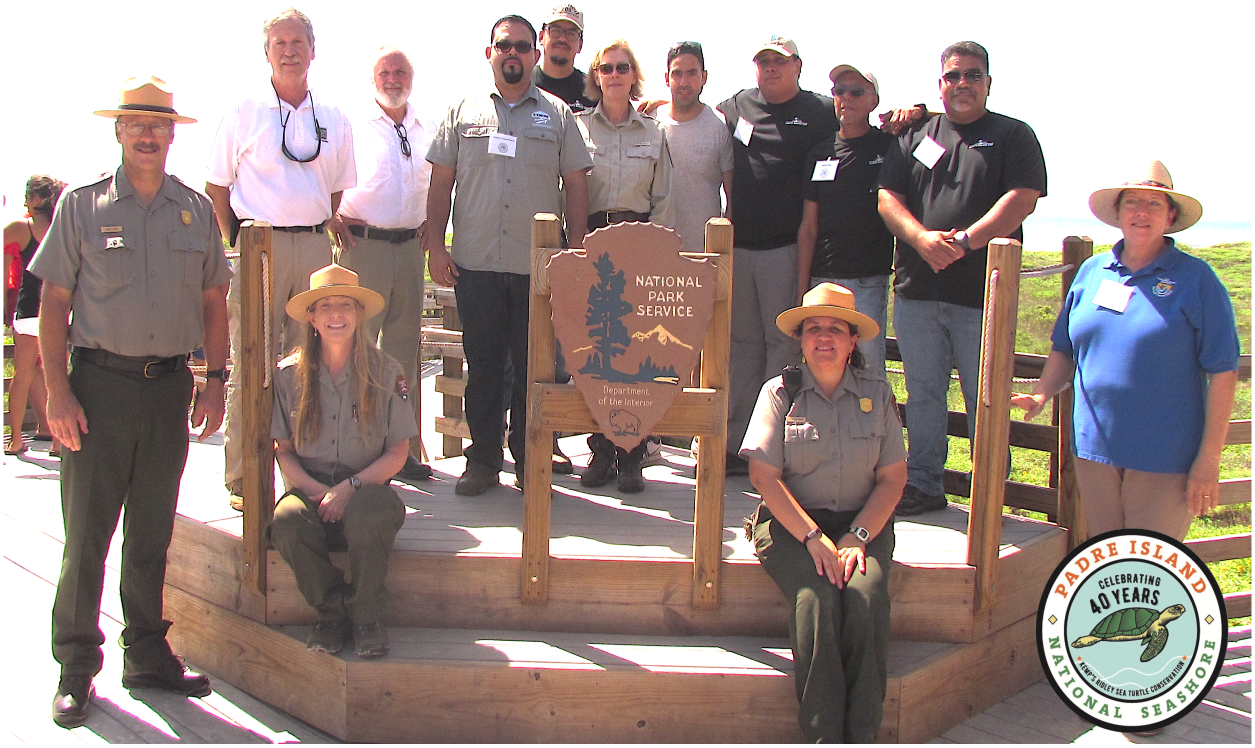

The Division of Sea Turtle Science and Recovery at Padre Island National Sea-shore (PAIS) celebrated 40 years of Kemp's ridley sea turtle conservation this weekend. The extensive cooperative efforts of the Kemp's Ridley Sea Turtle Restoration & Enhancement program have evolved into one of the most successful conservation stories to date. Conservation efforts began in 1978 when a bi-national partnership was launched to rescue a species on the brink of extinction — a species vital to the health of our ocean, the viability of our beaches, and now the heart of PAIS. Against all odds, and with the help of dedicated researchers, conservationists, and countless volunteers, this species went from producing 702 nests per year worldwide in 1985 (a 99.4% decline from historical records) to about 20,000 nests annually in recent years. This remarkable success story belongs to the Kemp's ridley sea turtle, the most endangered sea turtle species in the world.

A commemorative gathering, officially dubbed the "Ridley Rendezvous", kicked-off with a presentation at 6:30 am followed by a public hatchling release at 7:00 am. Additional events throughout the morning included interactive booths, mascot dance-off, and multiple Junior Ranger activities, as well as family-friendly presentations on various research projects devoted to Kemp's ridley sea turtles, presented by Dr. Donna Shaver and partners. At 10:15 am a second sea turtle release was held to release sea turtles that had been found stranded earlier this year, were rehabilitated back to health, and ready to be returned to the wild. This release included loggerhead, green, and Kemp's ridley sea turtles.

Local, state, federal, and international partners gathered to celebrate this milestone and demonstrate their continued support to Kemp's ridley sea turtle conservation. "I am thrilled by the wonderful support that has been shown by our partners and the community throughout the past 40 years," said Dr. Shaver. Dr. Patrick Burchfield, Director Gladys Porter Zoo and U.S. coordinator of the bi-national multiagency Kemp's Ridley Sea Turtle Restoration & Enhancement Project activities in Mexico, spoke at the event of the significance of the collaborative efforts. "It has taken a village… but if you give nature a chance, it *will* persist. I am honored to be a part of that village," Dr. Burchfield said.

Public response to the Kemp's ridley sea turtle conservation program also demonstrates the impact it has had on the community surrounding PAIS. The dedication of local volunteers contributing to the project and excitement exhibited by visitors during public hatchling releases are unparalleled. Although there is more work to be done to ensure the long-term survival of Kemp's ridley sea turtles, 40 years of cooperative conservation efforts are certainly paying off.

To learn more about the Division of Sea Turtle Science and Recovery at PAIS, please visit https://www.nps.gov/pais/learn/nature/stsr.htm. Also, there will be more opportunities to attend public hatchling releases through July if you have missed previous releases. Visit www.nps.gov/pais for an update on scheduled releases or call the Hatchling Hotline at 361-949-7163.

Felicia Page
Padre Island National Seashore
Division of Sea Turtle Science and Recovery
Felicia_Page@partner.nps.gov
